# [μ-*N*,*N*′-Bis(2-amino­eth­yl)ethane-1,2-diamine-κ^4^ *N* ^1^,*N* ^1′^:*N* ^2^,*N* ^2′^]bis­{[*N*,*N*′-bis­(2-amino­eth­yl)ethane-1,2-diamine-κ^4^ *N*,*N*′,*N*′′,*N*′′′]cadmium} tetra­kis­(perchlorate)

**DOI:** 10.1107/S1600536812033880

**Published:** 2012-08-04

**Authors:** Hamid Goudarziafshar, Yunes Abbasityula, Václav Eigner, Michal Dušek

**Affiliations:** a Faculty of Science, Department of Chemistry, Ilam University, Ilam, Iran; b Department of Solid State Chemistry, Institute of Chemical Technology, Technická 5, 166 28 Prague, Czech Republic; c Institute of Physics AS CR, v.v.i., Na Slovance 2, 182 21 Prague 8, Czech Republic

## Abstract

The centrosymmetric dinuclear cadmium title complex, [Cd_2_(C_6_H_18_N_4_)_3_](ClO_4_)_4_, was obtained by the reaction of *N*,*N*′-bis­(2-amino­eth­yl)ethane-1,2-diamine (trien) with Cd(NO_3_)_2_·4H_2_O and sodium perchlorate in methanol. The Cd^II^ cation is coordinated by four N atoms of a non-bridging trien ligand and by two N atoms of a bridging trien ligand in a slightly distorted octa­hedral coordination geometry. The bridging ligand shares another two N atoms with a neighboring symmetry-equivalent Cd^II^ cation. The structure displays C—H⋯O and N—H⋯O hydrogen bonding. The perchlorate anion is disordered over two sets of sites in a 0.854 (7): 0.146 (7) ratio.

## Related literature

Polyamines are an important class of *N*-donor ligands, particularly for transition metals, see: Patel *et al.* (2007 ▶); Blackman (2005 ▶). For polynuclear complexes, see: Gustafsson *et al.* (2010 ▶); Ambrosi *et al.* (2009 ▶); You *et al.* (2011 ▶). For polynuclear complexes of cadmium, see: Evans & Lin (2002 ▶); For background to the use of the trien ligand in complexation, see: Cai *et al.* (2001*a* ▶,*b* ▶); Buckingham *et al.* (1974 ▶, 1975 ▶); Chowdhury *et al.* (2007 ▶). Buckingham & Jones (1965 ▶); Shinohara *et al.* (1991 ▶); He (2009 ▶); Patel *et al.* (2008 ▶); Anderson *et al.* (1977 ▶); Shoukry *et al.* (1998 ▶); Hu *et al.* (2000 ▶). For dinuclear Cd complexes, see: Das *et al.* (2010 ▶); Nie *et al.* (2010 ▶); Wang *et al.* (2011 ▶); Sun *et al.* (2010 ▶). For details of the preparation, see: Harrowfield *et al.* (1996 ▶).
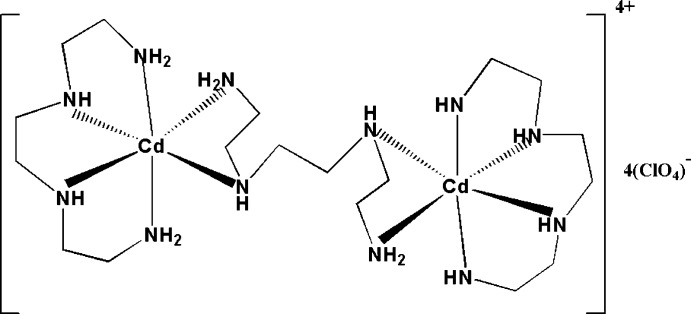


## Experimental

### 

#### Crystal data

[Cd_2_(C_6_H_18_N_4_)_3_](ClO_4_)_4_
*M* *_r_* = 1061.3
Monoclinic, 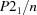

*a* = 8.8056 (2) Å
*b* = 15.0259 (3) Å
*c* = 14.7516 (3) Å
β = 95.4420 (17)°
*V* = 1943.02 (7) Å^3^
*Z* = 2
Mo *K*α radiation
μ = 1.45 mm^−1^
*T* = 120 K
0.70 × 0.51 × 0.33 mm

#### Data collection

Agilent Xcalibur Atlas Gemini ultra diffractometer
Absorption correction: analytical (*CrysAlis PRO*; Agilent, 2012 ▶) *T*_min_ = 0.509, *T*_max_ = 0.738
31295 measured reflections
4952 independent reflections
4373 reflections with *I* > 3σ(*I*)
*R*_int_ = 0.022

#### Refinement

*R*[*F* ^2^ > 2σ(*F* ^2^)] = 0.030
*wR*(*F* ^2^) = 0.106
*S* = 2.02
4952 reflections
269 parameters
H atoms treated by a mixture of independent and constrained refinement
Δρ_max_ = 1.13 e Å^−3^
Δρ_min_ = −0.92 e Å^−3^

### 

Data collection: *CrysAlis PRO* (Agilent, 2012 ▶); cell refinement: *CrysAlis PRO*; data reduction: *CrysAlis PRO*; program(s) used to solve structure: *SUPERFLIP* (Palatinus & Chapuis, 2007 ▶); program(s) used to refine structure: *JANA2006* (Petříček *et al.*, 2006 ▶); molecular graphics: *ORTEP-3 for Windows* (Farrugia, 1997 ▶) and *Mercury* (Macrae *et al.*, 2008 ▶); software used to prepare material for publication: *publCIF* (Westrip, 2010 ▶).

## Supplementary Material

Crystal structure: contains datablock(s) global, I. DOI: 10.1107/S1600536812033880/ru2040sup1.cif

Structure factors: contains datablock(s) I. DOI: 10.1107/S1600536812033880/ru2040Isup2.hkl

Additional supplementary materials:  crystallographic information; 3D view; checkCIF report

## Figures and Tables

**Table 1:** Selected bond lengths (Å)

Cd1—N1	2.362 (2)
Cd1—N4	2.390 (3)
Cd1—N7	2.377 (3)
Cd1—N10	2.380 (3)
Cd1—N11	2.374 (3)
Cd1—N14	2.375 (2)

**Table 2:** Hydrogen-bond geometry (Å, °)

*D*—H⋯*A*	*D*—H	H⋯*A*	*D*⋯*A*	*D*—H⋯*A*
C5—H1*c*5⋯O4*c* ^i^	0.96	2.49	3.449 (3)	172
N1—H2*n*1⋯O3*c* ^ii^	0.80 (4)	2.46 (4)	3.149 (4)	145 (3)
N11—H1*n*11⋯O2*c* ^iii^	0.82 (4)	2.40 (4)	3.140 (4)	149 (3)
N11—H2*n*11⋯O4*a* ^ii^	0.92 (4)	2.44 (4)	3.210 (5)	141 (3)
N4—H1*n*4⋯O4*a* ^ii^	0.98 (3)	2.36 (3)	3.271 (4)	154 (2)
N4—H1*n*4⋯O5*a* ^ii^	0.98 (3)	2.38 (3)	3.236 (4)	145 (2)
N4—H1*n*4⋯O5*b* ^ii^	0.98 (3)	2.22 (3)	3.113 (13)	151 (3)

Symmetry codes: (i) 
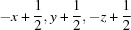
; (ii) 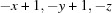; (iii) 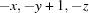.

**Fig. 1.**
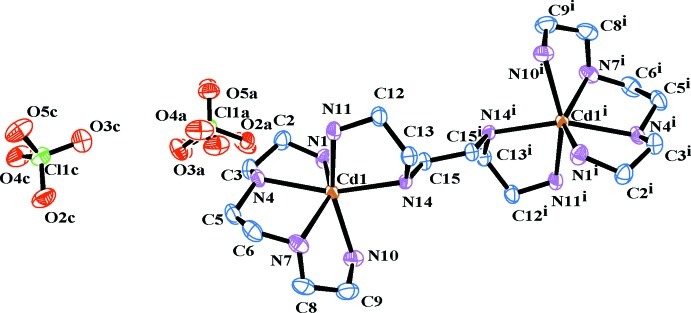
The molecular structure of the title compound with 50% probability displacement ellipsoids.

**Fig. 2.**
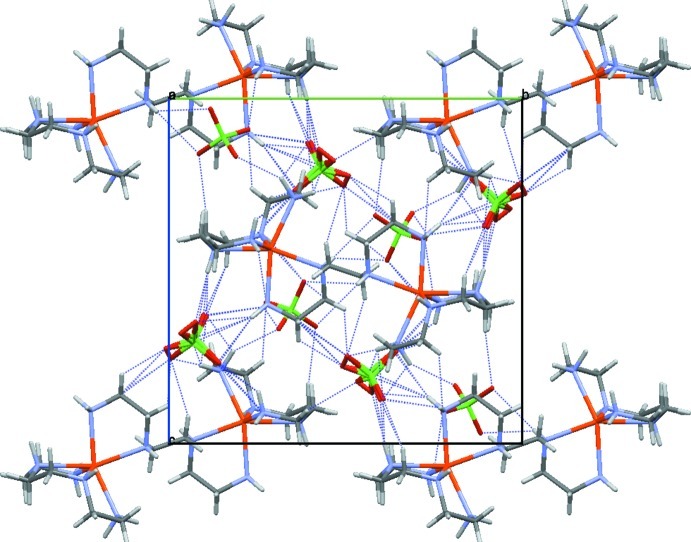
Unit-cell packing diagram of the title compound viewed along the crystallographic 〈〉a〈〉 axis. Hydrogen bonds are indicated by dashed lines; (orange = cadmium; green = chlorine; violet = nitrogen; grey = carbon; light-grey = hydrogen).

## supplementary crystallographic information

### Comment

Polyamines are an important class of N donor ligands, particularly for the transition metals (Patel *et al.*, 2007, 2008; Blackman, 2005). Considerable attention has been focused on the polynuclear complexes containing bridging ligands because of their interesting molecular topologies, as well as the fact that they may be designed with specific functionalities (Gustafsson *et al.* 2010;. Ambrosi *et al.* 2009; You *et al.* 2011). The investigation of polynuclear complexes of cadmium(II) is an important objective in view of their electronic and optoelectronic properties (Evans *et al.* 2002; Chowdhury *et al.* 2007).

The molecular structure of the title complex is shown in Fig. 1. The cadmium(II) centers are six-coordinate by four nitrogen atoms of the non-bridging tetradentate (trien) ligand and two nitrogen atoms of the bridging trien ligand, with a substantial departure from an ideal octahedral geometry [*cisoid* angles: 73.97 (8)–114.67 (9)°; *transoid* angles: 141.95 (1)–159.20 (6)°] (Table 1). The distance between the two cadmium(II) centers of the dinuclear complex is 7.735 Å, which is longer than the corresponding distance in dinickel(II) complex (7.497 Å) of the same ligand (Cai *et al.* 2001*b*) due to larger radius of cadmium. Cadmium atoms in the dinuclear complex are related by a 2 fold symmetry operation. Bond distance of Cd—N(trien) are in the range of 2.62 (3)- 2.90 (3) Å (Table 1). The structure exhibits disorder of one of the perchlorate anions in two positions with refined occupancy 0.854 (7) and 0.146 (7) for the major and minor componet,respectively. The disorder was described using the rigid body approach. In the title complex the C—H···O and N—H···O hydrogen bonds have been found between the amine nitrogen/carbon donors and perchlorate acceptors (Fig.2),(Table 2).

### Experimental

*N*,*N*'-bis(2-aminoethyl)ethane-1,2-diamine (0.45 g, 3 mmol) was placed in one arm of a branched tube (Harrowfield *et al.*, 1996) and a mixture of Cd(NO_3_)_2_.4H_2_O (0.616 g, 2 mmol) and sodium perchlorate (0.488 g, 4 mmol) in the other. Methanol was then carefully added to fill both arms, the tube sealed and the ligand-containing arm immersed in a bath at 333 K, while the other was left at ambient temperature. After one week, colorless crystals were collected in the cooler arm. Then they were filtered off, washed with acetone and diethylether, and air dried. Yield: (53%).

### Refinement

All hydrogen atoms were discernible in difference Fourier maps and could be refined to reasonable geometry. According to common practice H atoms bonded to C were kept in ideal positions with C–H = 0.96 Å while positions of other H atoms were refined freely. In both cases *U*_iso_(H) was set to 1.2*U*_eq_(C,*N*). Disorder of perchlorate anion was refined using rigid body refinement, with occupancy ratio 0.85:0.15.

### Crystal data

**Table:**

[Cd_2_(C_6_H_18_N_4_)_3_](ClO_4_)_4_	*F*(000) = 1076
*M**_r_* = 1061.3	*D*_x_ = 1.814 Mg m^−^^3^
Monoclinic, *P*2_1_/*n*	Mo *K*α radiation, λ = 0.7107 Å
Hall symbol: -P 2yn	Cell parameters from 17686 reflections
*a* = 8.8056 (2) Å	θ = 2.9–29.3°
*b* = 15.0259 (3) Å	µ = 1.45 mm^−^^1^
*c* = 14.7516 (3) Å	*T* = 120 K
β = 95.4420 (17)°	Prism, colourless
*V* = 1943.02 (7) Å^3^	0.70 × 0.51 × 0.33 mm
*Z* = 2	

### Data collection

**Table:**

Agilent Xcalibur Atlas Gemini ultra diffractometer	4952 independent reflections
Radiation source: Enhance (Mo) X-ray Source	4373 reflections with *I* > 3σ(*I*)
Graphite monochromator	*R*_int_ = 0.022
Detector resolution: 10.3784 pixels mm^-1^	θ_max_ = 29.4°, θ_min_ = 2.9°
ω scans	*h* = −11→12
Absorption correction: analytical (*CrysAlis PRO*; Agilent, 2012)	*k* = −19→20
*T*_min_ = 0.509, *T*_max_ = 0.738	*l* = −20→19
31295 measured reflections	

### Refinement

**Table:**

Refinement on *F*^2^	82 constraints
*R*[*F*^2^ > 2σ(*F*^2^)] = 0.030	H atoms treated by a mixture of independent and constrained refinement
*wR*(*F*^2^) = 0.106	Weighting scheme based on measured s.u.'s *w* = 1/(σ^2^(*I*) + 0.0016*I*^2^)
*S* = 2.02	(Δ/σ)_max_ = 0.045
4952 reflections	Δρ_max_ = 1.13 e Å^−^^3^
269 parameters	Δρ_min_ = −0.92 e Å^−^^3^
0 restraints	

### Special details

**Table:**

Experimental. Absorption correction: analytical: CrysAlisPro, Agilent Technologies, Version 1.171.35.19 Analytical numeric absorption correction based on crystal shape

### Fractional atomic coordinates and isotropic or equivalent isotropic displacement parameters (Å^2^)

**Table:**

	*x*	*y*	*z*	*U*_iso_*/*U*_eq_	Occ. (<1)
Cd1	0.286722 (18)	0.787742 (11)	0.064763 (11)	0.01848 (7)	
N1	0.5386 (3)	0.73266 (16)	0.08829 (18)	0.0272 (7)	
C2	0.5399 (3)	0.63634 (18)	0.0671 (2)	0.0292 (8)	
C3	0.4054 (3)	0.59042 (17)	0.10338 (19)	0.0283 (8)	
N4	0.2615 (2)	0.62928 (15)	0.06265 (14)	0.0228 (6)	
C5	0.1244 (3)	0.60375 (18)	0.10733 (18)	0.0300 (8)	
C6	−0.0058 (3)	0.6674 (2)	0.08095 (19)	0.0319 (9)	
N7	0.0352 (3)	0.76022 (18)	0.10385 (16)	0.0277 (7)	
C8	0.0284 (3)	0.78350 (18)	0.1997 (2)	0.0330 (9)	
C9	0.1157 (3)	0.8669 (2)	0.22447 (19)	0.0354 (9)	
N10	0.2773 (3)	0.85403 (16)	0.21066 (15)	0.0286 (7)	
N11	0.2454 (3)	0.77849 (15)	−0.09631 (18)	0.0296 (7)	
C12	0.2788 (3)	0.86355 (17)	−0.14108 (17)	0.0274 (8)	
C13	0.2345 (3)	0.94099 (17)	−0.08204 (17)	0.0231 (7)	
N14	0.3148 (2)	0.93478 (13)	0.00986 (14)	0.0183 (6)	
C15	0.4798 (3)	0.95387 (15)	0.01611 (16)	0.0200 (7)	
H1c2	0.632977	0.610336	0.094187	0.035*	
H2c2	0.535246	0.628241	0.002352	0.035*	
H1c3	0.407828	0.528128	0.089118	0.034*	
H2c3	0.411784	0.596978	0.168367	0.034*	
H1c5	0.147832	0.604608	0.172219	0.036*	
H2c5	0.094348	0.544411	0.089398	0.036*	
H1c6	−0.035184	0.662706	0.016775	0.0383*	
H2c6	−0.093123	0.650524	0.111322	0.0383*	
H1c8	0.068971	0.735436	0.2374	0.0396*	
H2c8	−0.076102	0.791229	0.211536	0.0396*	
H1c9	0.106623	0.881033	0.287172	0.0424*	
H2c9	0.074914	0.914972	0.18688	0.0424*	
H1c12	0.221103	0.866719	−0.199579	0.0329*	
H2c12	0.385736	0.866777	−0.14855	0.0329*	
H1c13	0.260242	0.996164	−0.109623	0.0277*	
H2c13	0.126385	0.940048	−0.07791	0.0277*	
H1c15	0.530028	0.909867	−0.017582	0.024*	
H2c15	0.523023	0.945412	0.077724	0.024*	
H1n10	0.331 (4)	0.819 (2)	0.267 (2)	0.0344*	
H2n10	0.340 (4)	0.909 (2)	0.227 (2)	0.0344*	
H1n1	0.574 (4)	0.746 (2)	0.148 (2)	0.0326*	
H1n7	−0.025 (5)	0.793 (2)	0.079 (3)	0.0332*	
H1n14	0.278 (3)	0.964 (2)	0.037 (2)	0.0219*	
H2n1	0.604 (4)	0.753 (3)	0.061 (2)	0.0326*	
H1n11	0.153 (5)	0.768 (2)	−0.104 (3)	0.0355*	
H2n11	0.285 (4)	0.736 (3)	−0.133 (3)	0.0355*	
H1n4	0.241 (3)	0.610 (2)	−0.001 (2)	0.0274*	
Cl1c	0.20589 (7)	0.15386 (4)	0.11243 (4)	0.03020 (19)	
O2c	0.0975 (3)	0.18921 (16)	0.16787 (16)	0.0431 (7)	
O3c	0.3065 (3)	0.22454 (16)	0.0906 (2)	0.0500 (9)	
O4c	0.2906 (3)	0.08460 (15)	0.16043 (15)	0.0451 (8)	
O5c	0.1347 (3)	0.11760 (15)	0.03083 (15)	0.0456 (7)	
Cl1a	0.8393 (2)	0.42448 (12)	0.19271 (14)	0.0246 (3)	0.854 (7)
O2a	0.8867 (6)	0.5086 (2)	0.2242 (3)	0.0768 (18)	0.854 (7)
O3a	0.8423 (5)	0.3641 (3)	0.2674 (3)	0.0378 (8)	0.854 (7)
O4a	0.6891 (4)	0.4247 (3)	0.1474 (3)	0.0468 (11)	0.854 (7)
O5a	0.9442 (5)	0.3933 (3)	0.1297 (3)	0.0388 (9)	0.854 (7)
Cl1b	0.8447 (14)	0.4319 (9)	0.2081 (8)	0.0246 (3)	0.146 (7)
O2b	0.9407 (15)	0.4949 (9)	0.2534 (9)	0.0768 (18)	0.146 (7)
O3b	0.8281 (14)	0.3574 (9)	0.2662 (9)	0.0378 (8)	0.146 (7)
O4b	0.6967 (14)	0.4661 (9)	0.1803 (9)	0.0468 (11)	0.146 (7)
O5b	0.9136 (14)	0.4022 (9)	0.1280 (9)	0.0388 (9)	0.146 (7)

### Atomic displacement parameters (Å^2^)

**Table:**

	*U*^11^	*U*^22^	*U*^33^	*U*^12^	*U*^13^	*U*^23^
Cd1	0.01761 (13)	0.01696 (13)	0.02150 (13)	−0.00220 (5)	0.00519 (8)	0.00130 (5)
N1	0.0215 (11)	0.0231 (11)	0.0368 (13)	−0.0034 (9)	0.0021 (10)	−0.0032 (10)
C2	0.0242 (13)	0.0229 (12)	0.0407 (15)	0.0027 (10)	0.0053 (11)	−0.0014 (11)
C3	0.0302 (14)	0.0206 (12)	0.0338 (14)	−0.0002 (10)	0.0015 (11)	0.0043 (10)
N4	0.0252 (10)	0.0207 (11)	0.0227 (11)	−0.0071 (8)	0.0028 (8)	0.0030 (8)
C5	0.0324 (14)	0.0275 (13)	0.0306 (14)	−0.0112 (11)	0.0050 (11)	0.0044 (11)
C6	0.0200 (13)	0.0419 (17)	0.0339 (14)	−0.0112 (11)	0.0023 (10)	0.0042 (12)
N7	0.0187 (11)	0.0357 (13)	0.0289 (12)	0.0004 (9)	0.0041 (9)	0.0065 (10)
C8	0.0249 (14)	0.0429 (17)	0.0329 (15)	0.0045 (11)	0.0116 (12)	0.0036 (11)
C9	0.0357 (15)	0.0384 (16)	0.0336 (15)	0.0088 (12)	0.0118 (12)	−0.0014 (12)
N10	0.0310 (12)	0.0322 (12)	0.0231 (11)	0.0017 (10)	0.0047 (9)	−0.0011 (9)
N11	0.0434 (15)	0.0194 (11)	0.0265 (12)	−0.0092 (10)	0.0066 (11)	−0.0021 (9)
C12	0.0395 (15)	0.0222 (12)	0.0200 (11)	−0.0088 (11)	0.0001 (10)	0.0006 (10)
C13	0.0214 (11)	0.0208 (12)	0.0263 (12)	−0.0018 (9)	−0.0014 (9)	0.0026 (9)
N14	0.0182 (10)	0.0148 (9)	0.0224 (10)	−0.0017 (7)	0.0045 (8)	−0.0016 (7)
C15	0.0189 (11)	0.0173 (11)	0.0234 (12)	−0.0026 (9)	−0.0001 (9)	0.0023 (9)
Cl1c	0.0298 (3)	0.0308 (3)	0.0297 (3)	0.0041 (2)	0.0011 (3)	−0.0042 (2)
O2c	0.0390 (12)	0.0416 (11)	0.0498 (14)	0.0074 (10)	0.0101 (10)	−0.0168 (11)
O3c	0.0589 (17)	0.0402 (13)	0.0531 (14)	−0.0106 (10)	0.0159 (12)	−0.0020 (11)
O4c	0.0614 (15)	0.0400 (13)	0.0329 (11)	0.0157 (10)	0.0003 (10)	−0.0007 (9)
O5c	0.0517 (14)	0.0461 (13)	0.0361 (11)	0.0195 (10)	−0.0106 (10)	−0.0111 (10)
Cl1a	0.0346 (4)	0.0174 (5)	0.0224 (8)	−0.0034 (3)	0.0062 (4)	−0.0039 (4)
O2a	0.140 (4)	0.0407 (17)	0.060 (3)	−0.055 (2)	0.064 (3)	−0.0326 (18)
O3a	0.0459 (16)	0.0401 (14)	0.0267 (10)	−0.0048 (11)	0.0004 (10)	0.0130 (10)
O4a	0.0390 (14)	0.066 (2)	0.0348 (16)	0.0216 (14)	0.0008 (11)	0.0038 (15)
O5a	0.0396 (18)	0.0376 (16)	0.0420 (13)	−0.0078 (15)	0.0184 (13)	−0.0130 (11)
Cl1b	0.0310 (4)	0.0229 (5)	0.0196 (8)	−0.0082 (3)	0.0014 (4)	−0.0039 (4)
O2b	0.104 (4)	0.098 (2)	0.031 (3)	−0.084 (2)	0.023 (2)	−0.0276 (19)
O3b	0.0424 (15)	0.0356 (14)	0.0350 (11)	−0.0036 (11)	0.0020 (10)	0.0163 (10)
O4b	0.0542 (15)	0.049 (2)	0.0374 (17)	0.0258 (13)	0.0073 (12)	0.0057 (15)
O5b	0.0368 (18)	0.0497 (16)	0.0314 (14)	−0.0109 (14)	0.0112 (13)	−0.0133 (11)

### Geometric parameters (Å, º)

**Table:**

Cd1—N1	2.362 (2)	C9—H1c9	0.96
Cd1—N4	2.390 (3)	C9—H2c9	0.96
Cd1—N7	2.377 (3)	N11—C12	1.481 (4)
Cd1—N10	2.380 (3)	N11—H1n11	0.82 (4)
Cd1—N11	2.374 (3)	N11—H2n11	0.92 (4)
Cd1—N14	2.375 (2)	C12—C13	1.526 (4)
N1—C2	1.481 (4)	C12—H1c12	0.96
N1—H1n1	0.93 (3)	C12—H2c12	0.96
N1—H2n1	0.80 (4)	C13—N14	1.471 (3)
C2—C3	1.512 (4)	C13—H1c13	0.96
C2—H1c2	0.96	C13—H2c13	0.96
C2—H2c2	0.96	N14—C15	1.475 (3)
C3—N4	1.471 (3)	N14—H1n14	0.70 (3)
C3—H1c3	0.96	C15—C15^i^	1.519 (3)
C3—H2c3	0.96	C15—H1c15	0.96
N4—C5	1.480 (4)	C15—H2c15	0.96
C5—C6	1.515 (4)	Cl1c—O2c	1.418 (3)
C5—H1c5	0.96	Cl1c—O3c	1.439 (3)
C5—H2c5	0.96	Cl1c—O4c	1.428 (2)
C6—N7	1.472 (4)	Cl1c—O5c	1.413 (2)
C6—H1c6	0.96	Cl1a—O2a	1.397 (4)
C6—H2c6	0.96	Cl1a—O3a	1.425 (5)
N7—C8	1.463 (4)	Cl1a—O4a	1.424 (4)
N7—H1n7	0.78 (4)	Cl1a—O5a	1.449 (5)
C8—C9	1.497 (4)	Cl1b—O2b	1.397 (18)
C8—H1c8	0.96	Cl1b—O3b	1.425 (19)
C8—H2c8	0.96	Cl1b—O4b	1.424 (17)
C9—N10	1.469 (4)	Cl1b—O5b	1.449 (19)
			
N11—Cd1—N14	73.97 (8)	C8—C9—H2c9	109.47
N4—Cd1—N10	114.67 (9)	N10—C9—H1c9	109.47
N1—Cd1—N7	141.90 (9)	N10—C9—H2c9	109.47
N4—Cd1—N14	159.20 (6)	H1c9—C9—H2c9	109.17
Cd1—N1—C2	109.72 (16)	C12—N11—H1n11	110 (2)
Cd1—N1—H1n1	107 (2)	C12—N11—H2n11	103 (2)
Cd1—N1—H2n1	120 (3)	H1n11—N11—H2n11	102 (3)
C2—N1—H1n1	114 (2)	N11—C12—C13	109.4 (2)
C2—N1—H2n1	105 (3)	N11—C12—H1c12	109.47
H1n1—N1—H2n1	102 (3)	N11—C12—H2c12	109.47
N1—C2—C3	110.5 (2)	C13—C12—H1c12	109.47
N1—C2—H1c2	109.47	C13—C12—H2c12	109.47
N1—C2—H2c2	109.47	H1c12—C12—H2c12	109.59
C3—C2—H1c2	109.47	C12—C13—N14	110.6 (2)
C3—C2—H2c2	109.47	C12—C13—H1c13	109.47
H1c2—C2—H2c2	108.47	C12—C13—H2c13	109.47
C2—C3—N4	110.3 (2)	N14—C13—H1c13	109.47
C2—C3—H1c3	109.47	N14—C13—H2c13	109.47
C2—C3—H2c3	109.47	H1c13—C13—H2c13	108.36
N4—C3—H1c3	109.47	C13—N14—C15	115.44 (19)
N4—C3—H2c3	109.47	C13—N14—H1n14	106 (3)
H1c3—C3—H2c3	108.58	C15—N14—H1n14	111 (2)
C3—N4—C5	115.0 (2)	N14—C15—C15^i^	114.62 (18)
N4—C5—C6	110.6 (2)	N14—C15—H1c15	109.47
N4—C5—H1c5	109.47	N14—C15—H2c15	109.47
N4—C5—H2c5	109.47	C15^i^—C15—H1c15	109.47
C6—C5—H1c5	109.47	C15^i^—C15—H2c15	109.47
C6—C5—H2c5	109.47	H1c15—C15—H2c15	103.78
H1c5—C5—H2c5	108.35	O2c—Cl1c—O3c	108.37 (16)
C5—C6—N7	112.1 (2)	O2c—Cl1c—O4c	109.59 (14)
C5—C6—H1c6	109.47	O2c—Cl1c—O5c	111.57 (14)
C5—C6—H2c6	109.47	O3c—Cl1c—O4c	110.20 (15)
N7—C6—H1c6	109.47	O3c—Cl1c—O5c	109.05 (16)
N7—C6—H2c6	109.47	O4c—Cl1c—O5c	108.05 (13)
H1c6—C6—H2c6	106.74	O2a—Cl1a—O3a	109.7 (3)
C6—N7—C8	114.6 (2)	O2a—Cl1a—O4a	112.9 (3)
C6—N7—H1n7	110 (2)	O2a—Cl1a—O5a	108.5 (3)
C8—N7—H1n7	102 (3)	O3a—Cl1a—O4a	108.2 (3)
N7—C8—C9	111.7 (2)	O3a—Cl1a—O5a	108.9 (3)
N7—C8—H1c8	109.47	O4a—Cl1a—O5a	108.6 (3)
N7—C8—H2c8	109.47	O2b—Cl1b—O3b	109.7 (11)
C9—C8—H1c8	109.47	O2b—Cl1b—O4b	112.9 (11)
C9—C8—H2c8	109.47	O2b—Cl1b—O5b	108.5 (11)
H1c8—C8—H2c8	107.19	O3b—Cl1b—O4b	108.2 (11)
C8—C9—N10	109.8 (2)	O3b—Cl1b—O5b	108.9 (11)
C8—C9—H1c9	109.47	O4b—Cl1b—O5b	108.6 (11)

Symmetry code: (i) −*x*+1, −*y*+2, −*z*.

### Hydrogen-bond geometry (Å, º)

**Table:**

*D*—H···*A*	*D*—H	H···*A*	*D*···*A*	*D*—H···*A*
C5—H1*c*5···O4*c*^ii^	0.96	2.49	3.449 (3)	172
C8—H2*c*8···O3*b*^ii^	0.96	2.48	3.412 (13)	163
N10—H1*n*10···O2*c*^ii^	1.06 (3)	2.24 (4)	3.191 (3)	149 (3)
N10—H2*n*10···O2*b*^iii^	1.01 (3)	2.32 (3)	3.268 (13)	156 (3)
N1—H1*n*1···O3*a*^iii^	0.93 (3)	2.25 (3)	3.018 (5)	139 (3)
N1—H1*n*1···O3*b*^iii^	0.93 (3)	2.22 (4)	3.004 (13)	141 (3)
N7—H1*n*7···O5*c*^iv^	0.78 (4)	2.25 (4)	2.999 (3)	159 (4)
N1—H2*n*1···O3*c*^v^	0.80 (4)	2.46 (4)	3.149 (4)	145 (3)
N11—H1*n*11···O2*c*^iv^	0.82 (4)	2.40 (4)	3.140 (4)	149 (3)
N11—H2*n*11···O4*a*^v^	0.92 (4)	2.44 (4)	3.210 (5)	141 (3)
N4—H1*n*4···O4*a*^v^	0.98 (3)	2.36 (3)	3.271 (4)	154 (2)
N4—H1*n*4···O5*a*^v^	0.98 (3)	2.38 (3)	3.236 (4)	145 (2)
N4—H1*n*4···O5*b*^v^	0.98 (3)	2.22 (3)	3.113 (13)	151 (3)

Symmetry codes: (ii) −*x*+1/2, *y*+1/2, −*z*+1/2; (iii) −*x*+3/2, *y*+1/2, −*z*+1/2; (iv) −*x*, −*y*+1, −*z*; (v) −*x*+1, −*y*+1, −*z*.
